# Texosome-based drug delivery system for cancer therapy: from past to present

**DOI:** 10.7497/j.issn.2095-3941.2015.0045

**Published:** 2015-09

**Authors:** Hamideh Mahmoodzadeh Hosseini, Raheleh Halabian, Mohsen Amin, Abbas Ali Imani Fooladi

**Affiliations:** ^1^Applied Microbiology Research Center, Baqiyatallah University of Medical Sciences, Tehran 1435916471, Iran; ^2^Department of Drug and Food Control, Faculty of Pharmacy, Tehran University of Medical Sciences, Tehran 1417653861, Iran

**Keywords:** Cancer therapy, texosome mimetic, tumor microenvironment, immunotherapy

## Abstract

Rising worldwide cancer incidence and resistance to current anti-cancer drugs necessitate the need for new pharmaceutical compounds and drug delivery system. Malfunction of the immune system, particularly in the tumor microenvironment, causes tumor growth and enhances tumor progression. Thus, cancer immunotherapy can be an appropriate approach to provoke the systemic immune system to combat tumor expansion. Texosomes, which are endogenous nanovesicles released by all tumor cells, contribute to cell-cell communication and modify the phenotypic features of recipient cells due to the texosomes’ ability to transport biological components. For this reason, texosome-based delivery system can be a valuable strategy for therapeutic purposes. To improve the pharmaceutical behavior of this system and to facilitate its use in medical applications, biotechnology approaches and mimetic techniques have been utilized. In this review, we present the development history of texosome-based delivery systems and discuss the advantages and disadvantages of each system.

## Introduction

The global rise of cancer outbreaks and consequent endeavor to find efficient and specific treatments are becoming hot topics in the field of cancer research. Common protocols such as chemotherapy, radiotherapy, and surgery are not efficient enough to meet all the needs in cancer eradication. Cancer recurrence, lack of sufficient efficacy on metastatic cancer, and emerging drug resistance usually occur after classic therapies. Therefore, devising new treatment strategies with both high efficiency and low toxicity is necessary and is the first priority in cancer field. Given that malfunction of the immune system, especially in a tumor microenvironment, leads to tumor growth and tumor progression^1^, cancer immunotherapy can be an option to stimulate the systemic immune system to combat tumor expansion^2^. Immune responses are modulated through immunotherapy strategies, causing specific removal of tumor cells and retarding metastases and stimulating memory immune cells against disease recurrence^3^^,^^4^. Recently, exosomes, which are natural nanovesicles, have been introduced as candidates for cancer immunotherapy. Exosomes are endosomal membranous vesicles with sizes ranging from 30 to 100 nm secreted by all kinds of mammalian cells into the extracellular microenvironment both in pathologic and physiologic conditions^5^. Moreover, exosomes have been isolated from biological fluids, such as serum and urine, and in the supernatant of cell cultures. Compared with normal cells, more exosomes are released from cancerous cells^5^^,^^6^ (details regarding exosome properties were reviewed by Hosseini *et al*.^5^).

**Figure 1:**
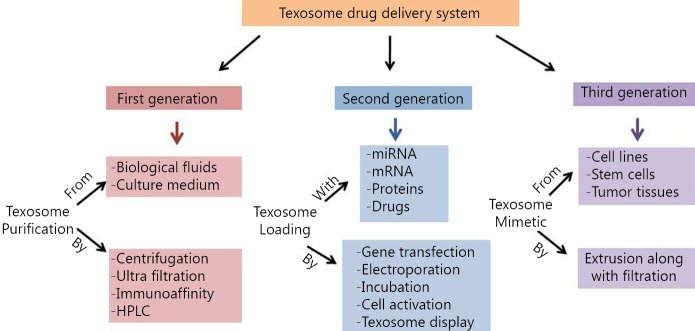
Schematic review for development history of texosome-based delivery system.

Exosomes exert several biological activities, including cell-cell communication and transport of genetic materials (e.g., miRNA and mRNA), alter the phenotypic characteristics of recipient cells via protein transport, and modulate the immune system^7^. The inherent potential of exosomes for delivering and carrying materials make them a suitable agent for drug delivery and gene therapy. In the past two decades, attempts have been made by researchers to understand the behavior of exosomes and their potency in drug delivery. To improve the exosomal drug delivery system, various manipulations have been conducted on intact exosomes, particularly on the mimetics of the exosomes (**Figure 1**). In this review, an overview of exosome drug delivery system is presented, and the classification of the system is explained in three categories: first-generation exosomes, in which the exosomes are applied without any manipulation; second-generation exosomes, in which biotechnological and bioengineering manipulations are applied; and third-generation exosomes, which are produced directly from cells through mimetic and synthetic methods.

## Exosome biogenesis

Exosomes are endogenous vesicles budding from endosome compartment during maturation of early endosome to late endosome as multi-vesicular bodies (MVBs)^8^. Evidence exists that the activity of phosphatidylinositol-3 kinase (PI-3 kinase) is essential to produce MVBs and in the subsequent secretion of exosomes in mammalian cells^8^^,^^9^. Loss of PI-3 kinase suppresses MVB formation due to endocytic compartment swelling^9^^,^^10^. In general, several factors determine the fate of MVBs. These factors include cholesterol content, presence of ligand for membrane proteins, proteins involved in the endosomal sorting complex required for transport (ESCART) system, tetraspanin proteins, and presence of sphingomyelinase. The cholesterol content of MVBs could fuse with the plasma membrane and secrete exosomes (cholesterol rich manner) or be digested after fusion with lysosome in poor cholesterol content^11^^,^^12^. Denzer *et al*.^8^ suggested that membrane proteins could incorporate into MVBs. Exosome biogenesis happens under two patterns, namely, ESCART-dependent or ESCART-independent pathways. Some accessory proteins, including Alix and vacuolar protein sorting 4 (VPS4), have been shown to be involved in the ESCART-dependent pathway^11^. Several processes have been explained in the ESCART-independent system. Sphingomyelinase, tetraspanin proteins, and certain regions such as endosome-like domains in the plasma membrane are involved in the independent pathway of exosome biogenesis^13^^-^^15^.

Finally, after the fusion of MVBs with the cellular membrane, the exosomes are secreted into the extracellular environment in both constitutive or inducible manner based on the type and condition of the cells^16^^,^^17^. Additionally, some members of the Rab family, such as Rab27a and b, participate in the exosome release^18^. Interestingly, soluble SNARE proteins can designate MVB for cellular membrane fusion^19^. Ubiquitination is one of the main mechanisms involved in the sorting of the endosomal proteins of MVBs. ESCRT proteins are necessary to move forward the MVB biogenesis. ESCRT-I, -II, and -III, Hrs, and Vps-27 are different kinds of protein complexes that recognize the monoubiquitinated cargos and lead them toward the MVB compartment. These complexes leave the MVB compartment with the help of adenosine triphosphatase and VPS4 after MVB formation and take part in the new cycle of cargo transportation^20^^,^^21^. Protein aggregation and clustering are two other mechanisms for sorting cargos. These mechanisms are independent of the monoubiquitination pathway^22^^,^^23^. Moreover, the passive manner for sorting cargos occurs via lipid raft-enriched tetraspanins and/or cholesterol^22^^,^^24^.

Exosomes target and bind to recipient cells selectively. This selectivity was confirmed in a study on the exosomes from platelets and B cells^25^. Moreover, exosomes from B cells attached only to follicular dendritic cells (DCs)^26^.

Similar to other fusion processes, the regulatory role of Ca^2+^ concentration and syntaxin-7 proteins make them plausible for exosome fusion^27^^,^^28^. Our findings of exosomal delivery to cells are limited. Results reported by Montecalvo *et al*.^29^ pointed out the potential of exosomes to transfer their cargos into the cytosol of targets. The interaction between exosomes and recipient cells is classified into three categories: (I) fusion through a subset of integrin family or via calcium and annexin V^26^^,^^30^^,^^31^; (II) ligand-receptor interplay^30^^,^^32^; (III) endocytosis^29^^,^^33^^,^^34^. The environmental factors and maturation level of cells regulate the turnover of exosome-recipient cell interactions as well as their fusion. Acidic environments increase the yield of fusion. Based on that finding, the fusion turnover within tumor tissues is higher than that in normal ones due to the acidic microenvironment of tumors^34^^,^^35^. Furthermore, maturation of bone marrow DCs reduced the uptake of exosomes^36^.

## Exosome structure and composition

Exosomes are bilayer membranous nanovesicles with endogenous origin. The composition profile of each exosome is closely related to the content of the cell of origin. Additionally, this profile dictates the functionality of exosomes^17^^,^^37^. Various common procedures, such as MALDI-TOF/Q-TOF mass spectrometry, trypsin digestion, immunoblotting, and SDS-PAGE, are utilized to appraise the protein content of exosomes. Moreover, thin layer chromatography is used for lipidomics analysis of exosome lipids^37^^-^^39^. The most diverse compounds within exosomes are proteins. Exosomal proteins are classified into two groups; the proteins found in all exosomes irrespective to the cells they are released from and those that are exclusive to a specific exosome. The proteins that are essentially involved in the exosomal biogenesis and functions are categorized in the first group^40^. This type of protein includes the ones contributing to membrane fusion, cytoskeleton components, cell-signaling molecules, adhesion proteins, chaperones, metabolic enzymes, MVB-forming proteins, and tetraspanin family proteins^37^^,^^41^. By contrast, the specific proteins within exosomes are proteins belonging to the cells from which the exosomes originate and contribute to certain roles dependent on the original cells^40^. For further information, please check the ExoCarta website^42^.

Limited research has been done on the lipidomic features of exosomes, and limited data are obtained on the lipid composition of exosomes obtained from some cells such as reticulocytes^43^, mast cells, DCs^44^, and B lymphocytes^24^. Sphingomyelin, lysophosphatidylcholine, saturated fatty acid, phosphatidyl ethanolamine, phosphatidylserine, phosphatidylcholine, diacylglyceride, and cholesterol are common lipids with different abundance detected in the membrane of exosomes derived from different origins^37^. Lysophosphatidic acid is a frequent lipid within exosomes and is necessary for exosome biogenesis and MVB formation^45^. Moreover, the functional units and micro-domains, i.e., lipid rafts, have been identified in exosome membranes. These units consist of major lipids, such as cholesterol and glycosphingolipids, and various proteins including Src family members and glycosyl-phosphatidylinositol-anchored proteins^46^.

Exosomes are considered genetic shuttles that are able to transfer genetic materials (e.g., mRNA and microRNAs) from the primary cells, conferring some new genetic and epigenetic features to the recipient cells^33^^,^^47^.

## Texosome roles in tumor progression

**Figure 2:**
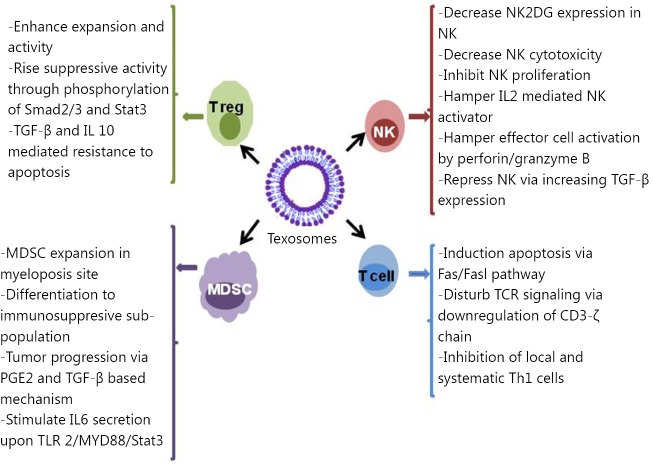
Effect of texosomes on the discrepant immune cells located in tumor microenvironment.

Tumor microenvironment is a space that determines the fate of tumor cells through communication among resident cells in tumor tissues, such as tumor cells themselves, immune cells, and matrix cells. **Figure 2** summarizes the effects of texosomes on the immune cells located in the tumor microenvironment. The release of inhibitory soluble substances suppresses the immune system located in tumor tissues and progresses the tumors^48^. One of the main agents contributing to this process is the exosome derived from tumor cells called texosome^49^^,^^50^. Convincing evidence exists for the presence of high quantity texosomes in blood and malignant effusions, which indicate the load and the stage of tumor in patients^51^^-^^53^. Carrying high amount of both known and unknown tumoral antigens, texosomes are offered as candidates for cancer vaccine^52^^,^^54^^,^^55^. However, numerous studies show the immunosuppressive effects of texosomes.

Texosomes can evade from the immune system through certain effects on both native and acquired immunity. Findings from studies on various cancer cell lines, including prostate cancer, head and neck cancer, gastric cancer, melanoma, and colorectal carcinoma, revealed that some texosomes trigger the expression of pro-apoptotic agents FasL and TRAIL^56^^-^^60^. These texosomes are able to interact with Fas molecules on the surface of active T cells and induce Fas/FasL apoptosis in T cells. In addition, pro-apoptotic texosomes cause CD3-ζ chain down-regulation and TCR signaling inhibition^61^. Increased adenosine level following dephosphorylation of 5’AMP and ATP via CD39 and CD73 happens during suppression of local immune responses^62^. In a study on natural killer (NK) cells, the cell function was impaired upon exposure to a texosome. This texosome prevented the expression of NK2GD in NK cells and subsequently attenuated their proliferation^63^^,^^64^. In another study, exposure of NK cells to texosomes containing MICA*008 alleviated the toxic effects of NK cells^65^. Moreover, the texosome derived from invasive murine breast tumor cells caused attenuated activation of NK cells following IL-2 secretion and decreased function of perforin/granzyme B-mediated effector^66^.

On the other hand, texosomes released by melanoma and colon carcinoma impaired the differentiation of CD14 monocyte to myeloid-derived suppressor cells, which repress activity and proliferation of T cells upon TGF-β release. This type of myeloid cell was isolated from blood of patients suffering from hepatocellular carcinoma^67^, bladder cancer^68^, and multiple myeloma^69^. Texosomes exerted the booster impacts on the activity and expansion of T regulatory cells (Treg) through phosphorylation of Stat3 and Smad2/3. These events led to the increase of the texosomes’ suppressing activity and resistance to TGF-β and IL-10 mediated apoptosis^70^.

In addition to suppressing the antitumor immune responses, texosomes are involved in tumor progression owing to improving angiogenesis and remodeling the extracellular matrix upon modulation of stromal cells and metastasis^71^.

Texosomes contain some functional proteins and genetic materials that take part in triggering the synthesis, formation, and expansion of extracellular matrix and vasculation^72^. Tetraspanins are essential components of MVB biogenesis and have been known as a pro-angiogenic factor that can incite tumor growth upon systemic angiogenesis^73^^,^^74^. The removal of Tspan8-CD49d complex from texosomes caused stimulation of gene expression of some angiogenic factors, such as Tspan8, von Willebrand factor, VEGF receptor 2, and VEGF. This complex induces and improves the proliferation, maturation, and migration of endothelial cell progenitors^75^. Moreover, Notch ligand delta-like 4 carrying texosomes contribute in vasculation and angiogenesis^76^. Overall, the presence of angiogenic factors, such as angiogenin, FGFa, IL-6, IL-8, TIMP-1, VEGF, and TIMP-2, in texosomes induces formation and improvement of tubular for vasculation^77^.

On the other hand, secretion of texosomes by cancer cells can give rise to resistance to chemotherapeutic drugs such as cisplatin and vinblastine through sequestering and pumping them out of the tumor cells. Resistance can also happen through the secretion of high amount of texosomes carrying transporters of drugs such as MRP2, ATP7A and ATP7B^78^. Furthermore, texosome secretion negatively affects the potency of antibody-based treatment^79^. This phenomenon occurs due to several mechanisms. The circulating texosomes in the peripheral blood bind to and neutralize the antibodies, thereby reducing the effective dose of antibodies against a tumor tissue. These texosomes attenuate antibody-dependent cytotoxicity of immune cells. Finally, secretion of texosomes has resulted in depletion of complement factors that protect tumor cells from antibody attack and inhibit cytolysis upon complement activation^80^^,^^81^.

## First generation of exosomal drug delivery system

Despite the tumorigenesis behavior of texosomes, these nanovesicles have properties that make them suitable for designing a noble anti-cancer vaccine. The presence of numerous broad-range tumor antigens [e.g., HER2, Mart-1, and carcinoembryonic antigen (CEA) along with MHC-peptide complexes within texosomes] confers a beneficial feature to T-cell cross-priming^52^^,^^82^^,^^83^. Several studies were performed based on this theory. **Table 1** outlines the pioneer studies in the field of texosome-based immunotherapy. Findings of these studies were the basis for future attempts for the design of efficient drug delivery systems.

**Table 1:** First-generation texosome-based delivery system for cancer therapy

Source of texosome	Treatment protocol	Type of cancer	Outcome	Ref
L1210 cell line	Vaccination with texosomes	Leukemia	Inhibitory impact on tumor growth, CTL mediated antitumor immunity	^84^
A20 (H-2d) B cell lymphoma/leukemia cell line, CT-26 colon adenocarcinoma cells	Vaccination with heat shocked texosome and texosome	Lymphoma, colon cancer, leukemia	CTL mediated antitumor immunity, heat stress produces texosome with higher efficiency against tumor	^85^
CT26 mouse colon carcinoma cells, B16-F1 mouse melanoma cells	Vaccination with dendritic cells pulsed with heat shock texosomes	Melanoma, colon cancer	Th1-mediated antitumor responses, rise IgG2a and IFN-γ production	^86^
CT26 and TA3HA mouse cell line	Vaccination with texosome derived from cell transfected with human MUC1	Colon cancer	Immune cell activation, inhibitory effect on growth of hMUC1-expressing tumor	^87^
Several pancreatic cancer cell lines	*In vitro* treatment with texosome	Pancreatic cancer	Stimulation of mitochondria mediated apoptosis and through p13k/Akt/GSK-3β	^88^
Soj-6 pancreatic cancer cell line	*In vitro* treatment with texosomes	Pancreatic cancer	Triggering apoptosis based on notch signaling	^89^
	Dendritic cells pulsed with texosome	Mesothelioma	Robust CTL mediated antitumor immunity	^82^

## Second generation of texosome drug delivery system

In 2011, Alvarez-Erviti and his colleagues^90^ conducted the first study on biotechnological manipulation of texosomes to make them applicable in targeted delivery of siRNA.

Texosome display strategy is another useful technology to create non-genetic engineering manipulation of texosomes for medical applications. In this strategy, a broad range of different antigens is fused with lipid bilayer compounds^91^^,^^92^. In this technology, antigens can be coupled with texosomes in a specific or non-specific binding manner. In specific fusion procedure, a recipient domain exists on the surface of texosomes that can attach to the desired antigen. For instance, in several studies, the presence of the C1C2 domain of lactadherin has been shown to be important for the fusion of antigens for therapeutic purposes^91^.

In non-specific method, the antigens are anchored in the membrane of texosomes, and the lumen is loaded with the components. Microbial metabolites and toxins, especially superantigens, can be used as cytostatic molecules in cancer prevention. Superantigens are potent T cell activators that can be suitable candidates for immunotherapy. These compounds attach to the major groove of MHC II proteins on the surface of antigen-presenting cells and enhance the proliferation and activation of T cells in a non-specific manner^93^. Previous researches showed that superantigens have the potential to trigger antitumor immunity^94^^-^^97^. Furthermore, the apoptotic features of superantigens via extrinsic pathway were reported^98^. Given that high amounts of tumoral antigens within texosome may induce energy in the immune system, designing a conjugate structure made up of texosomes and superantigens can activate the cytostatic events in tumor cells and can stimulate particular antitumor immune responses. The anchoring of staphylococcal enterotoxins A and B on tumor texosomes has been explained by Xiu *et al*.^99^ and Mahmoodzadeh Hosseini *et al*.^100^^-^^103^. Those studies are examples of the approach that confers an antitumor activity property to the construct. **Table 2** outlines the attempts using this strategy.

**Table 2:** Studies based on improved texosomal delivery system for cancer therapy

Texosome source	Modification method	Type of cargo	Type of cancer	Outcomes	Ref
HeLa, HT1080 human fibrosarcoma cell	Chemical treatment, electroporation	SiRNAs against RAD51 or RAD52	−	Effective post-transcriptional gene silencing, massive reproductive cell death	^104^
Breast cancer cell lines (HCC70, HCC1954, and MCF-7)	Gene transfection	let-7a, siRNA against EGFR, anti-EGFR peptide	Breast cancer	Inhibitory impact on the growth of breast cancer tumor model	^105^
Mouse immature dendritic cells	Gene transfection combined with electroporation	(Lamp2b) fused to αv integrin-specific iRGD peptide, doxorubicin	Breast cancer	Efficient targeting and delivery to tumor cells, suppression of tumor growth without obvious toxicity	^106^
EL-4	Incubation at 22 °C for 5 min	Curcumin and cucurbitacin I (Stat3 inhibitor)	GL26 brain tumor model	Prolong the growth of brain tumor, decrease the inflammation in brain, rising microglia cell apoptosis	^107^
HEK-293T cells	Gene transfection	Cytosine deaminase (CD) fused to uracil phosphoribosyltransferase (UPRT) (suicide gene)	Schwannoma tumors	Inhibition of tumor growth after treatment with modified exosomes accompanied with systematic administration of 5-FC	^108^
A murine melanoma cell line B16F1	Cell transduction to the CIITA gene (Class II transactivator)	High amount of MHC class II	Melanoma	Increase the proliferation of splenocyte and IL-2 release; enhance levels of TNF-α, chemokine receptor CCR7, and IL-12; delay tumor growth	^109^
J558 myeloma cell line	Gene transfection	Texosome contains membrane-bounded HSP70 pulsed with dendritic cells	Melanoma	CTL and NK cell-mediated antitumor responses	^110^
	Texosome display	Staphylococcal enterotoxin A anchored texosome	Lymphoma	Inhibitory impact on the growth of tumor; increase INF-γ and IL-2; prolong survival time	^99^
Marrow stromal cell	Transfection	Cel-miR-67 and hsa-miR-146b	9L gliosarcoma	M146-exo reduce tumor size	^111^
E.G7-OVA tumor cells	Transfection	Texosome containing IL-2	E.G7-OVA tumor cells	Induction of antitumor response by Th1 cells, CTL and NK cells, inhibitory effect on tumor growth	^112^
MCA101 C57Bl/6 fibrosarcoma	Transfection	Tumor antigen bounded to vesicle compared with soluble one	Fibrosarcoma	Inhibitory effect on tumor growth and induction antitumor immune response in bounded manner compared with soluble factor	^113^
MDA MB-231, MIA PACA-2, SKOV-3	Exosome display technology	Staphylococcal enterotoxin B anchored tumoral exosome	Breast cancer, pancreatic cancer, ovarian cancer	Induction of apoptosis via intrinsic pathway	^100^^-^^102^
Texosome mimetic					
U937cells, Raw 264.7 cells, and CT26 cells	Cell was extruded through 10, 5, and 1 μm filters	Chemotherapeutic drug-loaded texosomes	Colon adenocarcinoma	Dose-dependent TNF-α mediated cell death, inhibitory effect on tumor growth, and induction of antitumor immune responses	^114^
Embryonic stem cells	Extrusion through microchannels	Native mRNAs of Oct 3/4 and Nanog	NIH-3T3 fibroblasts	An efficient exosome mimetic method to deliver and express mRNA	^115^

## Methods for loading texosomes with therapeutic cargo

One strategy to alter the properties of texosomes and to give new characteristics to texosomes is the loading of different components within texosomes. Several techniques such as electroporation, transfection, and incubation are applied to load the texosomes^116^^,^^117^.

In electroporation, transient pores are created into texosomal membrane by an electrical field using 150-700 V, transferring the desired component across the membrane and reach the center of texosome lumen^118^. Previous studies utilized this technique for the uptake of siRNAs and doxorubicin^90^. Despite the success rate of this method to load various cargos, optimal parameters should be set based on the cell of origin^104^^,^^106^^,^^119^. In general, 0.07 to 0.5 μg/μL of texosomes is required for electroporation^90^^,^^106^^,^^116^^,^^120^. This method may be favorable in medical applications due to parameter control, but adverse effects may occur, such as loss of integrity of the texosome and the loaded cargo. In addition, some evidence reveals that electroporation can provoke the aggregation of both siRNAs and texosomes, which significantly reduce cargo preservation. However, optimizing the parameter and using some special media containing trehalose are able to attenuate texosome aggregates^116^^,^^117^.

Some transfection reagents such as HiPerFect and Lipofectamine 2000 are commercially available for loading siRNA into texosomes^104^^,^^119^. However, the efficacy of these reagents is lower than that of electroporation; therefore, it may not be an appropriate method for therapeutic purposes^104^^,^^119^. Another method for loading the therapeutic compounds is the isolation of texosomes from transfected cells containing overexpressed proteins of choice or miRNAs. These special products will be packaged into the secreted exosomes^105^^,^^111^. This strategy could be useful for tumor therapy to suppress certain oncogenes. The transfection-based method of loading cargo is an appropriate process. However, this method is not favorable for medical use when the individual donor cell was applied because the processes of achieving potent engineered cells are labor-intensive and time-consuming. An efficient transfected cell should produce texosomes bearing both targeting properties and containing high quality/quantity cargo.

Finally, certain incubation procedure can be utilized to load the desired cargo into texosomes. A study used this method to load curcumin into the lumen of texosomes for 5 min at 22 °C, inducing significant anti-inflammatory impacts in diseased models^107^^,^^121^. Curcumin can alter the fluidity of exosomal lipid bilayer and can facilitate movement of the cargo into the lumen^122^^,^^123^. Interestingly, 1 and 2 h incubation at 37 °C were successful for loading miR-150 and doxorubicin, respectively^114^^,^^124^. The size of the cargo is a key factor for its loading and movement across the membrane and has an impact on the efficacy of this method. In addition to loading technique, purification protocols improve the quantity and quality of texosome-based drug delivery systems for therapeutic purposes^125^. Limited research is available on the methods for loading cargos within texosomes. Therefore, novel procedures to improve loading efficiency are necessary for medical use.

## Third generation: texosome mimetics

Along with biotechnological strategies, synthesizing texosomes opened a new avenue to design an efficient texosomal drug delivery system named texosome mimetics. The idea of texosome mimetics has originated from the fact that numerous compounds existing in texosome structures, such as proteins and lipids, are unnecessary for special practical purposes. On the other hand, some components carried by natural texosomes are incompatible with therapeutic and medical purposes, and even some adverse effects may happen. Texosome mimetics provides the possibility to select special functional lipid, protein, and nucleic acid, such as siRNAs and miRNAs, as cargos according to desired purposes. Some structural similarities exist between texosomes and liposomes. Both of them have a spherical shape with size of lower than 100 nm, and their contents have been surrounded by lipid bilayer; thus, the principles involved in liposome preparation could be beneficial to texosome mimetics. These principles can provide a new field to generate efficient non-viral drug delivery systems^126^^,^^127^. The small size of texosomes allows them to penetrate the tissues and deliver the cargo (e.g., drugs) efficiently without systemic side effects. On the other hand, texosome mimetics improves the pharmacokinetic parameters, such as bioavailability, metabolism, and exertion. Mimetic texosomes can be classified based on the functional cargos and targeting components such as adhesion molecules or special ligands or receptors^114^. Cell extrusion by serial filtration is a procedure to synthesize exosome mimetics with structural properties relatively equivalent to natural texosomes. Data of several studies on texosome mimetics showed no side effects associated with different cell sources. Therefore, the application of non-autologous texosome mimetics is feasible for treating different diseases. In addition, similar to natural texosomes, texosome mimetics have the potential of loading and carrying several drugs (particularly chemotherapeutic and herbal drugs) to target cells without adverse effects on healthy cells. Surprisingly, all sorts of modifications discussed in the biotechnological manipulations and texosome display in the former sections can be applied in texosome mimetics to design new structures. This possibility is useful for dual targeting the structures needed for angiogenesis in cancer therapy. For this purpose, a drug delivery system with dual targeting (i.e., one toward cancerous cell and the other toward tumor endothelium) should be created to enhance the efficacy of antitumor activity, especially in drug-resistant tumors.

## Advantage of texosomal drug delivery system

Texosomes have specific characteristic of carrying functional materials within the body, which makes the texosomal delivery system a relatively new discipline for specific and efficient therapy. Texosomes are stable in blood circulation, especially against the activity of coagulant substances and complementary systems^90^^,^^128^, and their autologous usage is due to lack of immunogenicity^15^. In spite of considerable advantage over other delivery systems, the lower yield of texosome production for clinical application is an obstacle. Native texosomes form complex structures with unknown pharmaceutical properties^129^^,^^130^. Given that various endogenous vesicles from different cell types are present in biological fluids, the isolation of a specific population of the vesicle (e.g., texosome) is a difficult process or is even impossible. On the other hand, exogenous texosomes purified from a cell line are immunogens and trigger unpleasant immune response and adverse effects^131^.

To overcome the described disadvantages, texosome mimetics strategy was invented. In this strategy, texosomes are produced in large scale, which is desirable for preclinical or clinical applications. The yield of texosome production is 100 times higher than those of convenient purification methods. Texosome mimetics is a controllable process. The product is well characterized with explicit and acceptable pharmaceutical properties, and the impact of each substance can be studied^132^. The information concerning probable biological activities of functional proteins and lipids assembled in texosome mimetics is not accessible in the literature.

## Clinical trial

To date, few clinical trials have been conducted against cancer therapy. As outlined in **Table 3**, the first two accomplished studies on melanoma stage IIIb/IV^133^ and non-small cell lung cancer III/IV^134^ used the exosomes derived from the DCs of each patient. The exosomes were modified to present tumor antigens and were reinjected to the same patients. In another study on colorectal cancer stage III/IV, purified exosomes from ascites of each patient were administered along with GM-CSF^54^. All these phase I clinical trials emphasized on the desired immunostimulatory effects of exosome-based drug delivery systems in some patients, with no or minimal side effects. Recently, two clinical trials have been conducted based on herbal exosomes in colorectal cancer and head and neck cancer patients (**Table 3**). Overall, findings from previous trials confirm the feasibility of exosome in cancer therapy as a safe and specific approach.

**Table 3:** Clinical trials based on exosome delivery system for cancer therapy.

Drug	Effects	Disease	Status	Ref
Patient’s dexosome loaded with MAGE3	Rise the quantity of NK cells, expression of NKG2D in CD8^+^ and NK cells, minor toxicity, lack of specific CD8^+^ response	Melanoma stage IIIb/IV (phase I)	Completed	^133^
Patient’s dexosome loaded with MAGE3	Prolong disease stabilization, rise NK cell activity, minor toxicity, lack of specific CD8^+^ response	Non-small cell lung cancer III/IV (phase I)	Completed	^134^
Patient’s dexosome combined with chemotherapy (metronomic cyclophosphamide)	Higher immunostimulatory impact on T cells, suppress disease progression	Unresectable Non-small cell lung cancer	Completed	NCT01159288
Patient’s exosome combined with GM-CSF	Specific CD8^+^ response, no specific response after treating with exosome	Colorectal cancer stage III/IV (phase I)	Completed	^54^
Grape-derived exosomes	For attenuation of oral mucositis and pain related to chemotherapy and radiotherapy	Head and neck cancer	Ongoing	NCT01668849
Curcumin loaded exosome from the plant	For cancer treatment	Colorectal cancer	Ongoing	NCT01294072

## Conclusion

Recently, the trend in cancer therapy has shifted toward the design of biologically stable and safe delivery systems compatible with humans. The application of texosomes for safe and efficient immunotherapy opened a new window to cancer treatment. Despite the potential tumorigenesis properties of texosomes, the presence of a broad range of both known and unknown tumor antigens allowed the induction of significant antitumor immune responses. Moreover, texosome-based delivery system is stable without side effects. To improve this system, biotechnological approaches have been used to design a potent immunostimulatory delivery system capable of targeting properties. Mimetic technics allowed the engineering of texosome-based delivery systems in large scale to be used in medical applications. However, several subjects are still unclear in this discipline. In summary, our knowledge about the structure and function of the components, texosome mimetics, and their effects on each other is limited. Furthermore, the selection of proteins and lipids used in texosome assemblies is the key point in the field. Further studies are needed to address the current challenges of designing an efficient delivery system.
